# The burden of acute eye conditions on different healthcare providers in Wales: a retrospective population-based study

**DOI:** 10.23889/ijpds.v9i5.2557

**Published:** 2024-09-18

**Authors:** Anna Rawlings, Angharad Hobby, Barbara Ryan, Rachel North, Andrew Carson-Stevens, Mat Smith, Nik Sheen, Jennifer Acton

**Affiliations:** 1 Swansea University; 2 Cardiff University; 3 Univeristy of the West of England; 4 Health Education and Improvement Wales

## Objective & Approach

Demand for urgent eyecare exponentially outstrips public healthcare capacity in the United Kingdom. Such eye conditions, and related vision impairment, affect the lives of people everywhere. We aimed to quantify the burden of urgent eyecare on primary- and community-care providers in a national population using data produced by General Practitioner, Accident and Emergency, pharmacy and optometry services. A secondary aim was to characterise some drivers of this burden.

We analysed data from the Secure Anonymised Information Linkage Databank (linked), Eye Health Examination Wales Service (unlinked) and Common Ailments Scheme (unlinked) during 2017-2018 to quantify burden. We applied generalised linear modelling and logistic regression to understand drivers.

## Results

Of 365,044 episodes of care during the study, GPs and A&E managed 46.2% (168,877) and 1.4% (5,122) respectively while optometrists and pharmacists managed 51.8% (116,868) and 0.6% (2,635). Older females and infants of both sexes were more likely to use GP prescribing services, while adolescent and middle-aged males were more likely to visit A&E. GP prescribing burden was driven partially by economic deprivation, access to local services, and overall community health. Season, day of the week, and time of day were predictors of burden for GP and A&E services.

## Conclusion & Implications

Acute eyecare continues to place considerable burden on primary care services in Wales, particularly in urban areas with greater deprivation. This is likely to increase with an ageing population. With ongoing pathway development to better utilise community care, there may be scope to change this trajectory.

